# Inhibition of nuclear export restores nuclear localization and residual tumor suppressor function of truncated SMARCB1/INI1 protein in a molecular subset of atypical teratoid/rhabdoid tumors

**DOI:** 10.1007/s00401-021-02328-w

**Published:** 2021-05-18

**Authors:** Rajiv Pathak, Francesca Zin, Christian Thomas, Susanne Bens, Tenzin Gayden, Jason Karamchandani, Roy W. Dudley, Karolina Nemes, Pascal D. Johann, Florian Oyen, Uwe Kordes, Nada Jabado, Reiner Siebert, Werner Paulus, Marcel Kool, Michael C. Frühwald, Steffen Albrecht, Ganjam V. Kalpana, Martin Hasselblatt

**Affiliations:** 1 Departments of Genetics and Microbiology and Immunology, Albert Einstein College of Medicine, New York, NY 10461 USA; 2 Institute of Neuropathology, University Hospital Münster, Pottkamp 2, 48149 Münster, Germany; 3 Institute of Human Genetics, Ulm University & Ulm University Medical Center, Ulm, Germany; 4 Department of Human Genetics, McGill University, Montreal, Canada; 5 Department of Pathology, Montreal Neurological Institute, McGill University, Montreal, QC Canada; 6 Division of Neurosurgery, Department of Pediatric Surgery, Montreal Children’s Hospital, McGill University, Montreal, QC Canada; 7 Paediatric and Adolescent Medicine, Swabian Childrens’ Cancer Center, University Childrens’ Hospital Medical Center Augsburg and EU-RHAB Registry, Augsburg, Germany; 8 Hopp Children’s Cancer Center (KiTZ), Heidelberg, Germany; 9 Division of Paediatric Neurooncology, German Cancer Research Center (DKFZ) and German Cancer Consortium (DKTK), Heidelberg, Germany; 10 Department of Pediatric Hematology and Oncology, University Medical Center Hamburg-Eppendorf, Hamburg, Germany; 11 Division of Hematology/Oncology, McGill University, Montreal, QC Canada; 12 Princess Máxima Center for Pediatric Oncology, Utrecht, The Netherlands; 13 Department of Pathology, McGill University, Montreal, QC Canada

**Keywords:** Atypical teratoid/rhabdoid tumor, Malignant rhabdoid tumor, INI1, SMARCB1, BAF47, Cytoplasmic, Nuclear export signal, Selinexor

## Abstract

Loss of nuclear SMARCB1 (INI1/hSNF5/BAF47) protein expression due to biallelic mutations of the *SMARCB1* tumor suppressor gene is a hallmark of atypical teratoid/rhabdoid tumors (ATRT), but the presence of cytoplasmic SMARCB1 protein in these tumors has not yet been described. In a series of 102 primary ATRT, distinct cytoplasmic SMARCB1 staining on immunohistochemistry was encountered in 19 cases (19%) and was highly over-represented in cases showing pathogenic sequence variants leading to truncation or mutation of the C-terminal part of SMARCB1 (15/19 vs. 4/83; Chi-square: 56.04, *p* = 1.0E−10) and, related to this, in tumors of the molecular subgroup ATRT-TYR (16/36 vs. 3/66; Chi-square: 24.47, *p* = 7.6E−7). Previous reports have indicated that while SMARCB1 lacks a bona fide nuclear localization signal, it harbors a masked nuclear export signal (NES) and that truncation of the C-terminal region results in unmasking of this NES leading to cytoplasmic localization. To determine if cytoplasmic localization found in ATRT is due to unmasking of NES, we generated GFP fusions of one of the SMARCB1 truncating mutations (p.Q318X) found in the tumors along with a p.L266A mutation, which was shown to disrupt the interaction of SMARCB1-NES with exportin-1. We found that while the GFP-SMARCB1(Q318X) mutant localized to the cytoplasm, the double mutant GFP-SMARCB1(Q318X;L266A) localized to the nucleus, confirming NES requirement for cytoplasmic localization. Furthermore, cytoplasmic SMARCB1(Q318X) was unable to cause senescence as determined by morphological observations and by senescence-associated β-galactosidase assay, while nuclear SMARCB1(Q318X;L266A) mutant regained this function. Selinexor, a selective exportin-1 inhibitor, was effective in inhibiting the nuclear export of SMARCB1(Q318X) and caused rapid cell death in rhabdoid tumor cells. In conclusion, inhibition of nuclear export restores nuclear localization and residual tumor suppressor function of truncated SMARCB1*.* Therapies aimed at preventing nuclear export of mutant SMARCB1 protein may represent a promising targeted therapy in ATRT harboring truncating C-terminal *SMARCB1* mutations.

**Supplementary Information:**

The online version contains supplementary material available at 10.1007/s00401-021-02328-w.

## Introduction

Atypical teratoid/rhabdoid tumor (ATRT) is a highly aggressive central nervous system tumor mainly affecting infants [14]. Biallelic mutations of SWI/SNF chromatin remodeling complex member SMARCB1 (also known as INI1, hSNF5 or BAF47) are the characteristic genetic lesion and result in loss of nuclear SMARCB1 protein expression [10, 26]. While ATRT is a remarkably homogenous disease on the genetic level, DNA methylation and expression profiling studies have unveiled three molecular subgroups, i.e., ATRT-TYR, ATRT-SHH, and ATRT-MYC [20, 24, 38]. These subgroups not only show distinct DNA methylation profiles and gene expression signatures, but also differences in *SMARCB1* mutational patterns and clinical features [20]. ATRT-TYR, named after the enzyme tyrosinase that is highly expressed in this subgroup, often displays truncating C-terminal *SMARCB1* mutations as well as relatively favorable outcome [24, 25]. Molecular subgroup status has been recently shown to represent an independent prognostic factor [15, 40], but little is known on underlying biological processes that might explain clinical heterogeneity.

Protein function is tightly linked to intracellular location. Nuclear export of proteins involves interaction of leucine-rich nuclear export signals (NES) with exportin-1 (also known as CRM1) [13]. In tumor cells, exportin-1-mediated nuclear export has been shown to be upregulated and may cause cytoplasmic mis-localization of tumor suppressors, drug resistance and augmented tumor growth [17]. Specific inhibitors of nuclear export are being investigated for the treatment of hematologic malignancies and solid tumors [37], also including brain tumors such as glioblastoma [18].

SMARCB1 is an essential component of the SWI/SNF multiprotein complex that remodels the chromatin in an ATP-dependent manner [31]. SMARCB1 is a nuclear protein and harbors an N-terminal Winged Helix DNA binding domain, two highly conserved central domains that are imperfect repeats of each other known as Repeat (Rpt) 1 and 2 as well as a C-terminal coiled-coil domain [2, 32]. Cancer-associated mutations of *SMARCB1* are found in all domains of the protein, but many mutations affect the C-terminal region [12, 23]. Recent studies indicate that the SMARCB1 C-terminal domain harbors a basic α-helix structure that directly interacts with the nucleosome acidic patch [41]. While SMARCB1 is a nuclear protein, neither a nuclear localization signal (NLS) nor region/s of the protein responsible for nuclear localization are known at this point. However, previous studies have indicated the presence of a masked nuclear export signal (NES, amino acids 259–276) within the SMARCB1 Rpt2 region [9]. This NES region binds to exportin-1 via the conserved L266 residue [9]. In this study, it was demonstrated that truncation of the region C-terminus to the NES resulted in constitutive nuclear export and cytoplasmic accumulation of the truncated SMARCB1 protein. A mutation of NES residues (L266A) or treatment with nuclear export inhibitor Leptomycin-B prevented nuclear export and caused nuclear accumulation of the truncated protein [9]. Based on these results, it was proposed that the NES within the full-length protein was masked by the C-terminal region, and that truncation of this region leads to unmasking of NES and cytoplasmic accumulation of SMARCB1 C-terminal truncations [5, 9].

This study also described a *SMARCB1* mutation in an extracranial malignant rhabdoid tumor (c.delG950) that caused cytoplasmic accumulation of truncated protein and loss of tumor suppressor function in vitro [9]. However, c.delG950 mutations have not yet been described in ATRT, nor a systematic evaluation of ATRT samples for cytoplasmic SMARCB1 staining has been performed, and hence the relevance of the above observations for the biology of ATRT remained uncertain.

Here, we show that cytoplasmic accumulation of mutant SMARCB1 protein associated with truncating C-terminal *SMARCB1* mutations occurs in about 19% of ATRT, and that inhibition of nuclear export restores nuclear localization and residual tumor suppressor function of truncated SMARCB1 protein.

## Materials and methods

### ATRT samples

FFPE samples of 102 SMARCB1-deficient ATRT were retrieved from the archives of the Institute of Neuropathology Münster. The majority of samples had been collected in the context of the European Rhabdoid Tumor Registry EU-RHAB and include materials from 95 previously published cases (Table 1, for details see Supplemental Table 1) [15]. Ethics committee approval for the project was obtained (Ethics Committee of the University Hospital Münster 2009-532-f-S), and patients or the guardians gave informed consent for scientific use of archival materials. In all cases, the diagnosis of ATRT was confirmed using current WHO criteria. Furthermore, genetic characterization was performed and included *SMARCB1* sequencing, MLPA and FISH as described previously [15]. DNA methylation profiles were generated using the HumanMethylation450 BeadChip array or the Methylation EPIC BeadChip array (Illumina, San Diego, CA) and subjected to DNA methylation-based classification using the Heidelberg Brain Tumor Classifier (version v11b4) [6]. Fresh-frozen material of the Q318X mutant case could also be examined using whole genome sequencing and RNAseq.

**Table 1:** Patient characteristics and molecular findings in 102 ATRT samples

Age (median, interquartile range)	18 (10–28) months
Sex (male/female)	53/49
Tumor location	
Supratentorial	56 (55%)
Infratentorial	44 (43%)
Spinal	1 (1%)
Supra- and infratentorial	1 (1%)
SMARCB1 immunohistochemistry	
Loss of nuclear SMARCB1 staining	102 (100%)
Cytoplasmic SMARCB1 staining	19 (19%)
*SMARCB1* FISH	
Homozygous deletion	33 (32%)
Heterozygous deletion	44 (43%)
Wild type	25 (25%)
*SMARCB1* sequencing	
SNVs/indels present	49 (48%)
SNVs/indels absent	53 (52%)
Molecular subgroup	
ATRT-TYR	36 (35%)
ATRT-SHH	41 (40%)
ATRT-MYC	25 (25%)

### SMARCB1 immunohistochemistry

SMARCB1 immunohistochemistry was performed using a monoclonal antibody raised against amino acids 257–359 (BAF47; 1:200, BD Biosciences #612110) and a monoclonal antibody directed against amino acids 81–181; 1:200, Abcam ab58209) on an automated staining system (Dako Omnis, Agilent) as well as a monoclonal antibody directed against a C-terminal epitope (amino acids 350–385; 1:100, Abcam ab222519) on an automated staining system (Ventana BenchMark Ultra, Roche). Cytoplasmic SMARCB1 staining was rated independently by two blinded raters (MH and FZ) as distinct, faint or absent (for examples see Fig. 2b–d). In rare cases of discrepancy, staining results were jointly discussed until consensus was reached.

### Cell lines

293T cells (SMARCB1^+/+^) were propagated in Dulbecco’s modified Eagle’s medium (HyClone; Cat No: SH30081.01) supplemented with 1% Pen-Strep, 1% l-glutamate and 10% fetal bovine serum. MON cells derived from an extracranial rhabdoid tumor carrying a homozygous *SMARCB1* deletion (SMARCB1^−/−^) [42] were a gift of Dr. Olivier Delattre (Institut Curie, Paris, France) and were cultured in Roswell Park Memorial Institute (RPMI) medium (HyClone; Cat No: SH30096.01) supplemented with 1% Pen-Strep, 1% l-glutamate and 10% fetal bovine serum. When required, MON cells were selected with 500 µg/ml of Geneticin (Gibco; Cat. No. 10131-035).

### Site-directed mutagenesis to create Q318X substitution mutations and combinations

Site-directed mutagenesis (SDM) was performed using the QuikChange Lightning Site-Directed Mutagenesis kit (Agilent; Cat No: 210518) using XL10-Gold Ultracompetent Cells, as described by the manufacturer’s protocol. In order to create mutation at 318th position of SMARCB1 amino acids, SMARCB1_Q318 stop forward (5′-GCGAGCTCAGCTATCCCCGGATGCTGTA-3′) and SMARCB1_Q318 stop reverse (5′-TACAGCATCCGGGGATAGCTGAGCTGGC-3′) primers were used. We used both pEGFP- SMARCB1 and pEGFP-SMARCB1(L266A) plasmids as templates for site-directed mutagenesis, to create Q318X and (L266A;Q318X) mutations, respectively. After the mutagenesis and PCR amplification, the amplification products were digested with *DpnI* enzyme followed by the transformation into XL10-Gold ultracompetent cells, according to the manufacturer’s protocol. All mutants generated were confirmed by sequence analysis.

### Plasmids

All cloning was performed using the TOP10 strain of *Escherichia coli* unless noted otherwise. The plasmids pEGFP, pEGFP-SMARCB1, pEGFP-SMARCB1(L266A), pEGFP-SMARCB1(Q318X) and pEGFP-SMARCB1(L266A,Q318X) were isolated using Qiagen Endofree Plasmid Maxi kit (Qiagen; Cat No. 12362).

### Drugs used in the study

Leptomycin-B and Selinexor (KPT-330) were obtained from Santa Cruz (Cat No. 87081-35-4) and Selleckchem (Cat No. S7252), respectively. The stocks of 100 µg/ml Leptomycin-B (LMB) were prepared in 100% ethanol and 10 mM Selinexor (KPT-330) was prepared in 100% DMSO. The final working concentration for Leptomycin-B was 10 ng/ml and for Selinexor (KPT-330), it was 100 nM and 500 nM, respectively.

### Transfections and microscopy

Transfections were performed at 30–50% confluency, using 2 µg of endofree plasmid DNA per ml of respective growth medium within a 10 cm tissue culture dish. Briefly, 293T cells or MON cells were transfected with 20 µg of pEGFP-N1, pEGFP-SMARCB1, pEGFP-SMARCB1(L266A), pEGFP-SMARCB1(Q318X) or pEGFP-SMARCB1(L266A;Q318X) using Calcium phosphate transfection kit (Invitrogen; Cat No: K2780-01). A mixture of CaPO4-DNA precipitate was added drop by drop onto the cells. 293T cells were incubated for 16 h post addition of DNA. Media were changed and the cells were viewed at 18 h post-transfection. For MON cells, the complete growth medium was replaced by fresh c-DMEM at least 3 h before the transfection. CaPO4-DNA precipitate was added onto MON cells and incubated for 6 h. After the incubation, the medium was replaced by fresh C-RPMI medium and the cells were viewed 18 h post-transfection.

For the purpose of microscopy and immunostaining, 293T cells and MON cells were cultured and transfected on Lab-Tek II chambered slides (Thermo Scientific; Cat No: 177380). After transfection, the cells were fixed using Eddy fix (3.7% paraformaldehyde, 0.1% glutaraldehyde, 0.15 mg/ml saponin in PBS) for 15 min at room temperature, washed three times with phosphate-buffered saline (1 × PBS), and permeabilized with 0.5% Triton X-100 for 10 min at room temperature. Cells were mounted using ProLong Gold antifade reagent (Invitrogen; Cat No. P36934) and left overnight to settle at room temperature under dark. Next day, cells were imaged using a Leica SP5 Confocal Microscope and collected fluorescence data from 405 nm (DAPI-stained cell nuclei) and 488 nm (internalized labeled GFP signal).

### Senescent cell formation assay

MON cells at 40–50% confluency, plated on 6-well plates, were transfected with 5 µg of plasmids expressing GFP fusions of SMARCB1 or its mutants (pEGFP-N1, pEGFP-SMARCB1, pEGFP-SMARCB1(L266A), pEGFP-SMARCB1(Q318X) or pEGFP-SMARCB1(L266A;Q318X). After transfection, cells were cultured for 24 h prior to drug selection. Cells were maintained under 500 µg/ml of Geneticin selection for up to 13 days with several changes of media, prior to observation under the microscope. Transfections experiments were conducted three times independently. To quantitate morphologically distinct senescent cells, multiple (4–12) random fields of images were captured under the microscope (× 20 magnification) under the bright light, for each sample. The total number of cells and the number of senescent cells per each field were computed.

### Senescence β-galactosidase staining assay

MON cells were plated on 6-well plates at 40–50% confluency, and were transfected with 5 µg of plasmids each expressing GFP (pEGFP-N1) or GFP fusions of SMARCB1 or its mutants; SMARCB1(L266A), SMARCB1(Q318X) or SMARCB1(L266A;Q318X). Transfected cells were selected with 500 µg/ml of Geneticin for 13–14 days with several changes of media. On 13th (two experiments) or 14th day (one experiment), Senescence β-Galactosidase Cell Staining was performed according to the manufacturer's protocol (Cell Signaling Technology; Cat No: 9860) with some minor modifications. Briefly, cells were washed once with 1X PBS followed by fixation in β-galactosidase staining fix solution for 15 min at room temperature. Cells were then washed three times with 1X PBS and incubated with 1 ml of β-gal staining solution (Cell Signaling Technology; Cat No: 9860) for 4, 8 or 24 h. (for three different experiments) at 37 °C. After incubation, cells were washed with 1 × PBS and overlaid with 70% glycerol. Cells were observed under Zeiss Axio Observer CLEM (Correlative Light and Electron Microscopy). Three independent experiments were conducted with different times of incubation with β-galactosidase stain. All experiments yielded similar results and hence were included in the analysis. To quantitate β-Galactosidase positive cells, multiple random fields of images were captured (10–15 fields per experiment) under the microscope (× 20 magnification), and a minimum total of 200 cells were counted for each sample. The total number of cells and the number of β-gal-positive cells per each field were computed to determine the % of β-gal-positive cells.

### MTS cell proliferation assay

MON cells were transfected with plasmids expressing GFP, GFP fusions of SMARCB1 or its mutant SMARCB1(Q318X) in 10 CM plates. 24 h post-transfection, cells were harvested using trypsin and were transferred to 96-well plates at a density of about 20,000 transfected cells per well in 200 μl of medium. Cells were selected by adding 500 µg/ml of Geneticin. 24 h after transferring to 96-well plates, the cells were treated with 100 nM and 500 nM of Selinexor (KPT-330), respectively. The cells were monitored for survival using MTS Cell Proliferation Assay Kit (abcam; Cat No: ab197010) at 0, 4, 7 and 10 days post-treatment. To perform the assay, 20 μl of MTS Reagent was added into each well and incubated for 3 h at 37 °C in standard culture conditions. After 3 h, the absorbance of treated and untreated cells were determined at OD = 490 nm using PerkinElmer VICTOR Nivo Multimode plate reader. The values were expressed as % of untreated control.

### Statistics

Statistical analyses of cell culture data were carried out using Kruskal–Wallis test with Dunn’s multiple comparisons using GraphPad Prism. Survival analyses were performed using IBM SPSS Statistics (Version 26.0).

## Results

### Cytoplasmic SMARCB1 staining occurs in a substantial proportion of ATRT

**Fig. 1:**
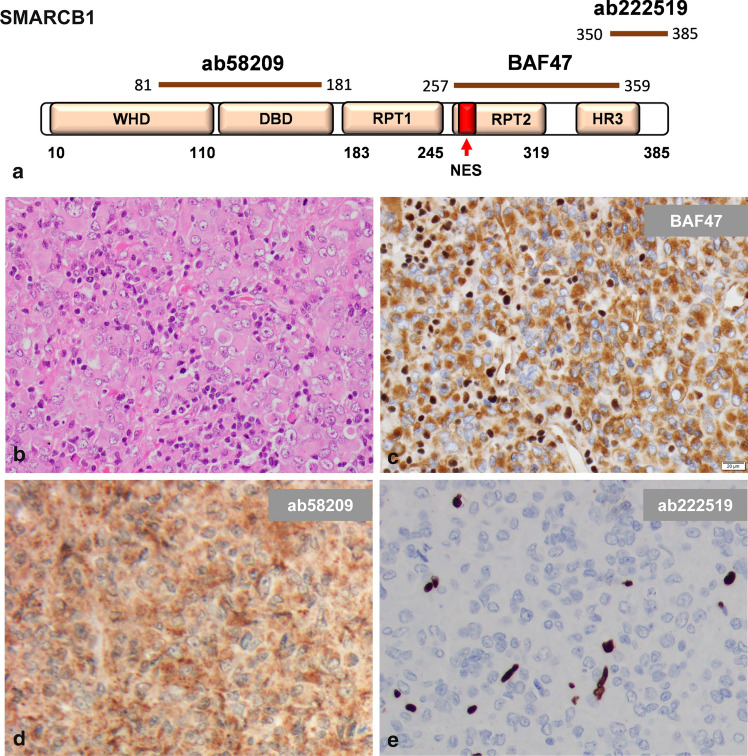
Cytoplasmic SMARCB1 staining in ATRT. Immunohistochemistry was performed using three different antibodies directed against N-terminal and C-terminal epitopes (**a**). Representative ATRT with rhabdoid tumor cells (**b**) showing strong cytoplasmic SMARCB1 staining using the BAF47 antibody (**c**) as well as another antibody directed against a more N-terminal epitope (ab58209, **d**), but absent cytoplasmic staining when using an antibody directed against the C-terminus of SMARCB1 (ab222519, **e**). In this representative case, a truncating C-terminal *SMARCB1* mutation was identified (p.Q318X). Note that all antibodies yield negative nuclear staining of tumor cells, while non-neoplastic cells show retained nuclear staining (internal positive controls). *WHD* Winged Helix domain; *DBD* DNA binding domain; *RPT* Repeat; *NES* Nuclear Export Signal; *HR3* homology region 3 (coiled-coil domain)

In a series of 102 ATRT samples (Table 1), distinct cytoplasmic SMARCB1 staining was encountered in 19 cases (19%) using immunohistochemistry and a commercial mouse monoclonal antibody (BAF47) raised against amino acids 257–359 of SMARCB1 (ENST00000644036.2), which is commonly used in the diagnostic setting [19, 26, 27] (Fig. 1a–c). In these cases, cytoplasmic staining was especially encountered in tumor cells showing rhabdoid or epithelioid morphology, i.e., tumor cells with more abundant cytoplasm. Similar results were obtained when using an antibody directed against a more N-terminal epitope of SMARCB1 (amino acids 81–181, ab58209, Fig. 1d). In contrast, using an antibody directed against the C-terminus of SMARCB1 (amino acids 350–385, ab222519), all cases that had shown distinct cytoplasmatic staining using the BAF47 antibody displayed negative cytoplasmatic staining (Fig. 1e). All antibodies yielded negative nuclear staining of tumor cells, while non-neoplastic cells show retained nuclear staining (internal positive controls). These results suggest the presence of cytoplasmic C-terminally truncated SMARCB1 protein.

### Cytoplasmic SMARCB1 staining is related to C-terminal SNVs/indels

**Fig. 2:**
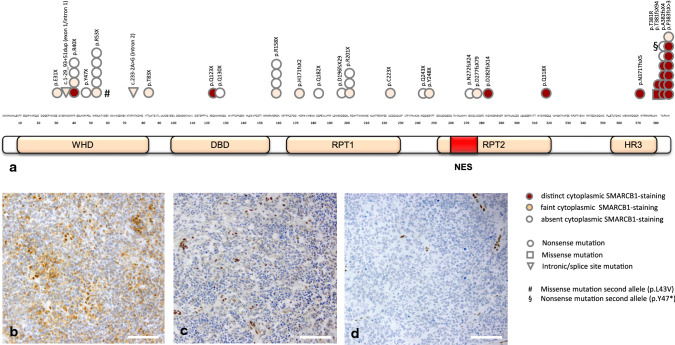
Cytoplasmic SMARCB1 staining status according to SMARCB1 mutation. Immunohistochemical staining results using the BAF47 antibody in 49 ATRT, in which *SMARCB1* SNVs/indels were encountered (**a**). Note that distinct cytoplasmic staining is highly over-represented in cases showing SNVs/indels C-terminal of the nuclear export sequence (NES). The majority of the SNVs/indels were nonsense (circles) and only one missense (square) and two intronic mutations (triangles) were encountered. # Missense mutation of the second allele (p.L43V), § Nonsense mutation of the second allele (p.Y47X). *WHD* Winged Helix domain; *DBD* DNA binding domain; *RPT* Repeat; *NES* Nuclear Export Signal; *HR3* homology region 3 (coiled-coil domain). Representative staining examples for distinct (**b**), faint (**c**) as well as absent cytoplasmic SMARCB1 staining (**d**) are also given

Cytoplasmic SMARCB1 staining using the BAF47 antibody was observed in 17/49 cases in which *SMARCB1* SNVs/indels were demonstrated on sequencing, but only 2/20 cases harboring *SMARCB1* deletions affecting both alleles detected by MLPA and FISH and 0/33 cases showing larger homozygous deletions affecting the *SMARCB1* region detected by FISH (Chi-square 16.88, *df* = 2, *p* = 2.2E−04, for details, see Supplementary Table 1). Interestingly, distinct cytoplasmic SMARCB1 staining was highly over-represented in cases showing SNVs/indels C-terminal of the NES [15/19 (78.9%) vs. 4/83 (4.8%); Chi-square: 51.27, *p* = 1.0E−10, Fig. 2] and of the molecular subgroup ATRT-TYR, in which C-terminal *SMARCB1* mutations are common [16/36 (44.4%) vs. 3/66 (4.5%); Chi-square: 24.47; *p* = 7.6E−7]. Most of the SNVs/indels were predicted to truncate the protein. In addition, a likely pathogenic missense mutation (p.T381R) and two mutations affecting splice sites also present in the germline were encountered. Large heterozygous deletions of the second allele represented the second hit in the majority of cases. In three of the cases showing distinct cytoplasmic SMARCB1 staining of tumor cells, germline SNVs/indels C-terminal of the NES could be demonstrated. Here, cytoplasmic SMARCB1 staining (albeit to lesser extent) was also observed in non-neoplastic cells. In this retrospective cohort, the Kaplan–Meier estimate for overall survival was 25 months (median; 95% confidence interval: 17–33 months) and the presence of distinct cytoplasmic SMARCB1 staining per se did not significantly affect overall survival (Log-Rank *p* = 0.37).

Taken together, these results indicate that cytoplasmic localization of truncated SMARCB1 protein occurs in a substantial proportion of ATRT harboring C-terminal SNVs/indels and that it has no prognostic role.

### Cytoplasmic accumulation of SMARCB1(Q318X) mutant protein is due to unmasking of the nuclear export sequence

Even though wild-type SMARCB1 is of nuclear location, it harbors a nuclear export signal (NES) within the Rpt2 region [9]. We have previously shown that truncating the C-terminal region of SMARCB1 leads to cytoplasmic localization of the mutant protein [9], likely due to unmasking of the nuclear export sequence [9]. Based on these observations, we hypothesized that cytoplasmic localization of truncated SMARCB1 protein in ATRT could be due to nuclear export sequence unmasking, which in turn may cause loss of tumor suppressor function.

**Fig. 3:**
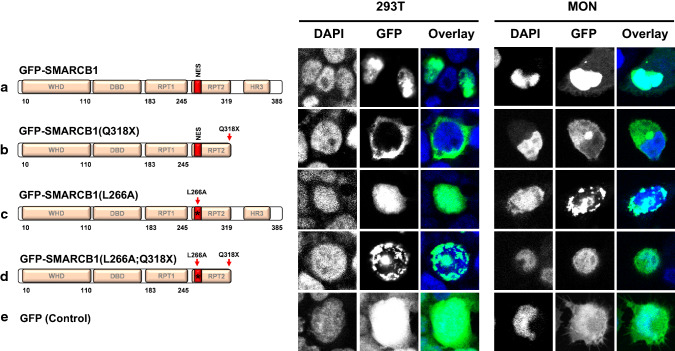
Effect of truncating mutations and NES disruption on sub-cellular localization of SMARCB1: Confocal imaging of 293T (*SMARCB1*^+*/*+^) and MON cells (*SMARCB1*^*−/−*^) showing nuclear localization of GFP-SMARCB1 (**a**), and cytoplasmic location of GFP-SMARCB1(Q318X) (**b**). While disruption of the NES does not alter nuclear localization of GFP-SMARCB1(L266A) (**c**), disruption of the NES in GFP-SMARCB1(L266A;Q318X) double mutant restores its nuclear localization (**d**). GFP-Control (**e**). Images were taken at 63 × (zoom-2.0) and in each row, the left panel shows nuclear DAPI staining, the middle panel GFP fluorescence, and the right panel the overlay of the two

We, therefore, analyzed the sub-cellular localization of GFP fusions of SMARCB1 harboring one of the truncating mutations (Q318X) along with a L266A mutation, which disrupts the NES, in vitro using two cell lines, 293T (*SMARCB1*^+/+^) and MON (*SMARCB1*^*−/−*^) cells. In both of these cells, GFP-SMARCB1 was localized in the nucleus (Fig. 3a), but the truncating Q318X mutation [(GFP-SMARCB1(Q318X)] caused a dramatic cytoplasmic accumulation of truncated protein (Fig. 3b). While disruption of the NES [(GFP-SMARCB1(L266A)] had no effect on sub-cellular localization (Fig. 3c), the double mutant [(GFP-SMARCB1(Q318X;L266A)] caused relocation of protein to the nucleus (Fig. 3d). Similar results were obtained in SMARCB1-deficient MON cells (Fig. 3a–d; right panel). These results are consistent with the hypothesis that cytoplasmic accumulation of Q318X requires a functional NES and that truncation of the C-terminal region in Q318X leads to NES unmasking.

### Inhibition of nuclear export restores nuclear location and residual function of truncated SMARCB1/INI1 protein

**Fig. 4:**
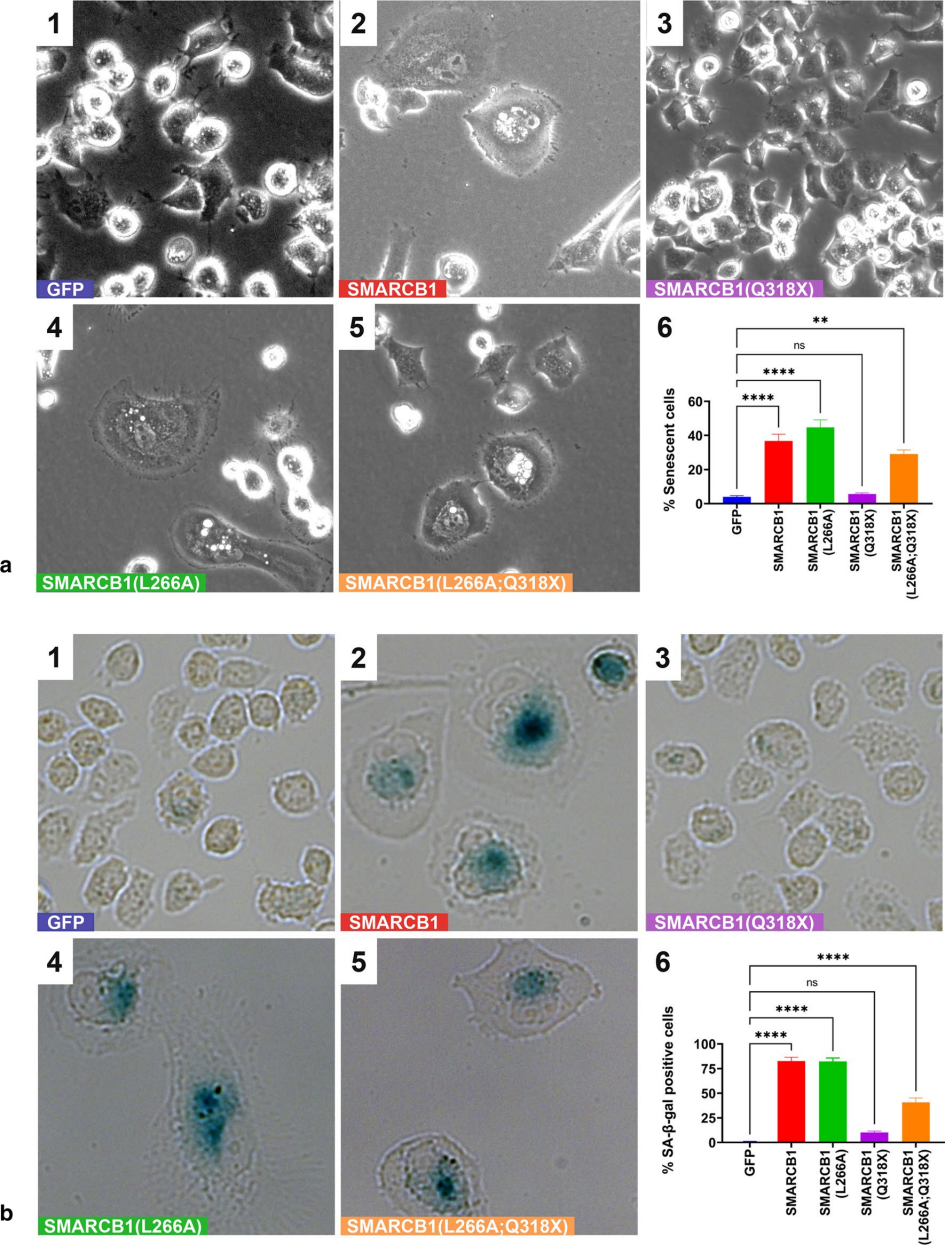
Functional effects of sub-cellular location of truncated SMARCB1: Senescent cell formation (**a**) and induction of SA-β-gal activity (**b**), indicative of senescence by SMARCB1 and mutants. Upon transfection in MON (SMARCB1^−/−^) cells, SMARCB1 as well as SMARCB1(L266A) increases the percentage of senescent cells and SA-β-gal-positive cells, while SMARCB1(Q318X) (i.e., truncated protein shown to be of cytoplasmic location) does not induce senescent cells or SA-β-gal-positive cells. In contrast, disruption of the NES in SMARCB1(L266A;Q318X) double mutant (shown to restore nuclear location of truncated protein) significantly induces senescent cells that are positive for SA-β-gal staining. **a** The senescent cell images were captured at 20X using the phase contrast setting. **b** The cells were stained with SA-β-gal and the images were captured after 13 days at 20 × using the Zeiss Axio Observer CLEM (Correlative Light and Electron Microscopy). Each experiment was performed three independent times and a representative image per sample is shown. Panels in **a** and **b** represent images of MON cells transfected with: GFP (panel 1); GFP-SMARCB1 (Panel 2); GFP-SMARCB1(Q318X) (Panel 3); GFP-SMARCB1(L266A) (Panel 4); and GFP-SMARCB1(Q318X;L266A) (Panel 5). Panel 6 represents the Graphical representation of the quantitation of data using multiple sets of transfection experiments indicating % of senescent cells (**a**); or % SA-β-gal-positive cells (**b**). (mean ± SEM. ****p value < 0.0001, **p value < 0.01, *ns* not significant)

To demonstrate that nuclear export of Q318X mutant results in inactivation of SMARCB1 tumor suppressor function, we carried out an SMARCB1-mediated senescent cell formation assay in MON cells. In this assay, expression of SMARCB1 results in the formation of large, flat and mitotically arrested cells indicative of senescence, which are easily distinguishable from actively dividing cells. To test the effect of Q318X mutation, GFP-SMARCB1 or GFP-SMARCB1 mutants were transfected into MON cells and selected for G418 (neomycin) resistance. Expression of GFP-SMARCB1 induced senescent cells as compared to GFP alone, while cytoplasmically localized GFP-SMARCB1(Q318X) was unable to induce senescent cell formation (Fig. 4a panels 1–3). However, the combination of L266A and Q318X mutations that caused nuclear localization of the truncated protein also restored its ability to induce senescent cells (Fig. 4a, panels 5). Quantitation of senescent cells from multiple experiments indicated that while wild type, L266A and (Q318X;L226A) mutants induced significant levels of senescent cells (*p* < 0.0001 for wild type and L266A mutants and *p* = 0.0069 for Q318X;L226A mutant), the percentage of senescent cells was not significant in Q318X mutant cells when compared to GFP transfected control cells (Fig. 4, panel 6).

To confirm the induction of senescence by SMARCB1 and lack of induction by SMARCB1(Q318X) mutant in MON cells, we stained the transfected cells for the expression of senescence-associated β-galactosidase (SA-β-gal). The results indicated that flat cells (but not the other cells) were clearly positive for SA-β-gal staining (Fig. 4b, panels 1–5). Quantitation of cells (*n* > 200 per sample in three independent experiments) for SA-β-gal-positive staining indicated that while wild type, L266A and the nuclear localized Q318X;L226A mutant induced significant levels of SA-β-gal-positive cells (*p* < 0.0001), the percentage of SA-β-gal-positive cells was not significant in cells expressing cytoplasmically localized Q318X mutant (*p* = 0.1124) when compared to GFP transfected control cells (Fig. 4b, panel 6). These results indicate that the inability of GFP-SMARCB1(Q318X) mutant to induce senescence is likely due to its cytoplasmic localization and that preventing nuclear export can restore residual tumor suppressor function of truncated SMARCB1 protein.

### Drugs that inhibit nuclear export also inhibit growth of rhabdoid tumor cells

**Fig. 5:**
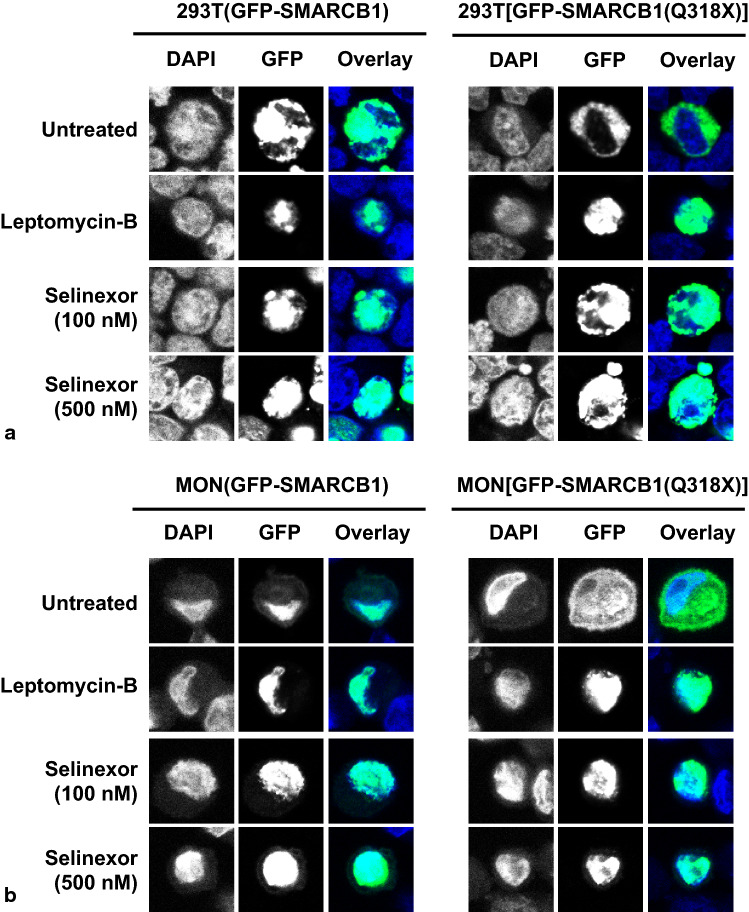
Selinexor (KPT-330) and Leptomycin-B restore the nuclear localization of SMARCB1(Q318X): Confocal imaging showing the effect of LMB and Selinexor (KPT-330) on sub-cellular location of GFP-SMARCB1 and GFP-SMARCB1(Q318X) in 293T (*SMARCB1*^+*/*+^, **a**) and in MON cells (*SMARCB1*^*−/−*^, **b**). Note that in both cell lines treatment with Leptomycin-B and Selinexor restores nuclear location of the GFP-SMARCB1(Q318X) mutant, indicating that both drugs effectively inhibit the nuclear export of the SMARCB1(Q318X) mutant. Images were taken at 63 × (zoom-2.0) and in each row, the left panel shows nuclear DAPI staining, the middle panel GFP fluorescence, and the right panel the overlay of the two

The above studies provided evidence for the hypothesis that reverting cytoplasmic localization of C-terminal truncations of SMARCB1 might provide a novel therapeutic avenue. Selinexor (KPT-330) is a selective inhibitor of nuclear export used as an anti-cancer drug [3, 36, 43] and was granted accelerated approval by the U.S. Food and Drug Administration (FDA) for treatment of multiple myeloma [33]. First, to test if Selinexor can inhibit nuclear export of SMARCB1(Q318X), we treated 293T cells (SMARCB1^+/+^) and MON (SMARCB1^−/−^) cells expressing GFP-SMARCB1 or the panel of mutants with Selinexor (KPT-330) followed by confocal microscopy. Leptomycin-B, a naturally occurring compound derived from bacteria that inhibits nuclear export by covalently modifying and, thus, inactivating exportin-1 was used as a control for nuclear export inhibition [28, 29]. Briefly, the two cell lines were treated with 10 ng/ml of Leptomycin-B and 100 nM and 500 nM of Selinexor (KPT-330), respectively. Concentrations of Selinexor were chosen based on the activity in other tumor cell lines [3, 16]. Six hours post-treatment, neither Leptomycin-B nor Selinexor affected the nuclear localization of GFP-SMARCB1. In contrast, treatment with Leptomycin-B and Selinexor resulted in nuclear location of the GFP-SMARCB1(Q318X) mutant, indicating that both drugs effectively inhibit the nuclear export of the SMARCB1(Q318X) mutant (Fig. 5).

**Fig. 6:**
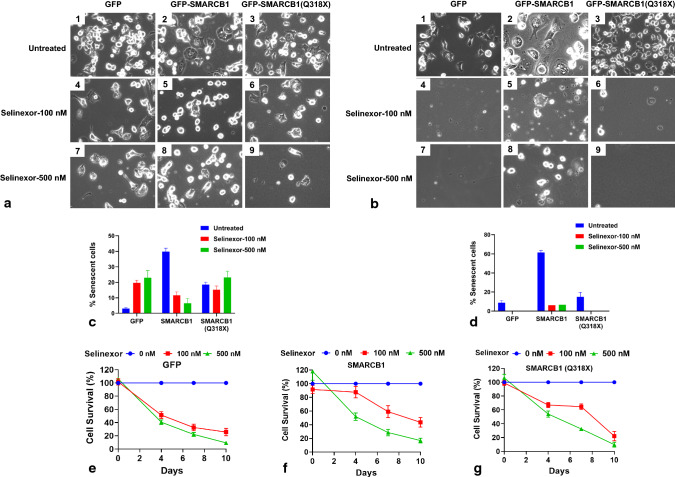
Effect of Selinexor (KPT-330) on cell growth and senescent cell formation in the presence and absence of SMARCB1 and SMARCB1(Q318X): Phase contrast microscopic visualization of senescent cell formation in MON (*SMARCB1*^*−/−*^) cells transfected with GFP, GFP-SMARCB1 or GFP-SMARCB1(Q318X) in response to Selinexor (KPT-330) 7 days (**a**) or 10 days (**b**) post-treatment. Images were captured at 20 × using the phase contrast setting. Shown are representative images. **c** and **d** Percentage of senescent cells per field of view of treated and untreated cells in *a* and *b*, Mean ± SEM. **e**–**g** Effect of Selinexor on cell survival. MON (*SMARCB1−/−*) cells transfected with GFP, GFP-SMARCB1 or GFP-SMARCB1(Q318X) were subjected to MTS cell proliferation assay at 0, 4, 7 and 10 days post-treatment with 100 or 500 nM Selinexor (% of treated compared to untreated, mean ± SEM)

Next, we aimed to determine if inhibition of nuclear export by Selinexor was sufficient to cause senescent cell formation and/or cell death in cells expressing GFP-SMARCB1(Q318X). We transfected GFP-SMARCB1 and GFP-SMARCB1(Q318X) into MON cells and selected for transfected cells by treating the cells with neomycin in the presence and absence of Selinexor (KPT-330). It has been observed that it takes ~ 7–13 days for SMARCB1 to induce senescent cell formation under these conditions [46]. In the absence of drug, SMARCB1 readily induced senescent cell formation in transfected cells, whereas cytoplasmically localized GFP-SMARCB1(Q318X) was unable to induce senescent cell formation as before (Fig. 6a, panels 1–3 and 6b, panels 1–3). Two distinct effects were noted upon Selinexor treatment with regard to senescent cell formation and cell death. By day 7, in the presence of Selinexor, percentage of senescent cells induced by SMARCB1(Q318X) was increased, consistent with the idea that Selinexor inhibited the cytoplasmic localization of this mutant protein (Fig. 6a, panels 6, 9 and 6c). Interestingly, Selinexor also induced cell death in both control cells and in transfected cells. MTS assay was carried out at 0, 4, 7 and 10 days after the addition of Selinexor and % cell death in treated cells as compared to untreated cells were determined (Fig. 6e–g). We found that Selinexor readily induced cell death (> 90%) by 10 days in cells not expressing SMARCB1 (Fig. 6e). Interestingly, 100 nM Selinexor was less effective in inducing cell death in cells expressing SMARCB1 and > 40% of the cells were alive by day 10 (Fig. 6f). Cells expressing Q318X mutant were also resistant to cell death by 100 nM Selinexor by day 7 (Fig. 6g). However, by day 10, growth of all the cells expressing or not expressing SMARCB1 or SMARCB1(Q318X) were inhibited to > 90% by 500 nM Selinexor treatment (Fig. 6e, f). These results suggest that Selinexor induces senescent cell formation by SMARCB1(Q318X), likely by inhibiting cytoplasmic localization, and that it is additionally effective in inhibiting growth of these cancer cells at higher concentrations.

Taken together, the cell culture data indicate that C-terminally truncated SMARCB1 mutants that are cytoplasmically localized due to the unmasking of nuclear export, appear to maintain residual tumor suppressor function when reverted back to the nucleus. This opens up the possibility for novel therapies for ATRT that show cytoplasmic accumulation of truncated SMARCB1.

## Discussion

The key findings of the present study are the observation that cytoplasmic accumulation of truncated SMARCB1 protein occurs in about 19% of ATRTs and that inhibition of nuclear export restores nuclear location and residual tumor suppressor function of truncated SMARCB1 proteins. These findings are not only of biological interest, but also have potential therapeutic implications.

Loss of nuclear SMARCB1 staining is the diagnostic hallmark of ATRT, but the presence of cytoplasmic SMARCB1 staining has never been systematically addressed and (if encountered) probably regarded as non-specific background staining. However, the finding that cytoplasmic SMARCB1 staining was highly enriched in cases showing C-terminal mutations and only encountered when using antibodies directed against N-terminal epitopes, strongly suggests that cytoplasmic accumulation of truncated SMARCB1 protein occurs in ATRTs harboring C-terminal *SMARCB1* mutations. In the diagnostic setting, this finding could give a first hint of the presence of C-terminal *SMARCB1* mutations and might aid the selection of subsequent molecular genetic studies and therapies. The few cases showing C-terminal mutations that lacked cytoplasmatic staining encountered in this retrospective series are most likely due to the use of archival materials that had been obtained from various institutions over a long time period. The same holds true for two cases in our series showing N-terminal mutations and non-recurrent cytoplasmic SMARCB1 staining, even though other mechanisms that may cause cytoplasmic location of SMARCB1 cannot be entirely excluded. In schwannomatosis, exon 1 mutations and re-initiation of translation associated with mosaic SMARCB1 staining pattern have been described [21].

We have previously shown that cytoplasmic localization of mutant SMARCB1 protein may be due to unmasking of an NES within the Rpt2 region [9]. While it is clear that nuclear export of truncated protein is mediated by binding to exportin-1, the mechanism by which the NES is masked by the region C-terminus to it is unknown at this point. We speculated that it is either due to binding of a cellular factor or due to folding of the C-terminal region such that the NES is blocked [9]. It is intriguing that recent structural studies indicate the presence of coiled-coil region at the C-terminus that binds to the acidic patch on the nucleosomes [43]. It is possible that binding of the C-terminal region to the nucleosome may be responsible for nuclear retention of the full-length protein, and lack of this binding may lead to nuclear export. Future studies to investigate this function are likely to shed light on the mechanism of SMARCB1 nuclear export.

It is interesting to note that the Q318X mutant is as defective as the absence of SMARCB1 with regard to inducing senescence. This defect in senescence is restored in a statistically significant manner, when a second mutation is introduced in the NES region (L266A). Interestingly, the ability of the double mutant (Q318X;L226A) to cause senescence is slightly reduced. One of the reasons could be that the double mutant may be less potent in its tumor suppressor function, which is of interest for the future studies. Another reason could be that the double mutant is expressed to a lower level in some cells in these transient transfection assays and may not be sufficient to induce senescence in these cells, leading to a reduction in the percentage of senescent cells. Nevertheless, the fact that the double mutant significantly induces senescent cells compared to that of the single mutant supports our hypothesis that the Q318X mutant has retained some tumor suppressor function, which is expressed when it is redirected to the nucleus.

In human cell lines, shuttling of full-length SMARCB1 between the nucleus and the cytoplasm has been demonstrated [1]. SMARCB1 protein in the cytoplasmic compartment was shown to interact with a number of proteins including dynamin-2 [1], which is a GTPase involved in endocytosis and vesicle dynamics. Of note, the interaction with dynamin-2 involved C-terminal domains of SMARCB1 [1]. SMARCB1/INI1 is also an important host factor for HIV-1 replication and it is incorporated into HIV-1 virions [45]. Furthermore, when HIV-1 virus infects a target cell, the nuclear export of SMARCB1 was stimulated in the cells being infected [39]. However, the functional role of SMARCB1 nuclear export in HIV-1 replication is not completely understood at this point. In *Drosophila* it has been shown that Snr1, the fly homologue of SMARCB1, is not only of nuclear but also of cytoplasmic location and that cytoplasmic Snr1 exerts tumor suppressive roles by affecting endosomal trafficking of membrane proteins [44].

The findings of the present study extend the observations of Craig et al. [9] where it was suggested that nuclear export of C-terminally truncated SMARCB1 could be associated with tumorigenesis. The current results suggest that this holds also true for C-terminal truncating mutations encountered in ATRT. In contrast to extracranial malignant rhabdoid tumors, which characteristically show homozygous deletions affecting the *SMARCB1* region [8], C-terminal truncating mutations are frequent in ATRT and predominantly encountered in the molecular subgroup ATRT-TYR [20]. The overrepresentation of cytoplasmic SMARCB1 staining in cases of the molecular subgroup ATRT-TYR thus probably reflects the high prevalence of C-terminal truncating mutations in ATRT-TYR [20] and not an inherent feature of this molecular subgroup. Furthermore, cytoplasmic accumulation of mutant SMARCB1 protein was not associated with overall survival, suggesting that a relatively favorable outcome of ATRT-TYR [15] is probably rather related to other biological and/or clinical features of this molecular subgroup and not to the cytoplasmic presence of truncated SMARCB1 protein.

The SWI/SNF complex exerts its biological actions and tumor suppressor role in the nucleus [31]. In many cancers, aberrant cytoplasmic localization of tumor suppressors plays a functional role. In breast cancer, for example, cytoplasmic mislocalization of BRCA1 is frequent [7] and has been linked to tumor aggressiveness [34]. Our finding that cytoplasmically localized C-terminally truncated SMARCB1 protein failed to induce senescence in vitro, argues against a tumor suppressor role of truncated SMARCB1 in the cytoplasm This view is further supported by the observation that cytoplasmic SMARCB1 staining per se did not affect overall survival in children with ATRT, which had not received treatment with nuclear export inhibitors. Disruption of the NES, however, not only restored nuclear location of truncated SMARCB1 protein, but also induced senescence in the senescent cell assay and SA-β-gal assay, suggesting residual tumor suppressor function of the truncated protein, if nuclear location can be achieved.

Selective inhibition of nuclear export may restore normal tumor suppressor function and represents a promising approach for the treatment of cancer [22, 35]. While Leptomycin-B was one of the first natural compounds to be used to inhibit nuclear export and tumor growth, its toxicity has prevented further utilization of this compound for clinical use [11].

Selinexor (KPT-330) is an advanced selective inhibitor of nuclear export [3, 36, 43] that recently received FDA approval for treatment of multiple myeloma [33]. Selinexor has also been shown to slow tumor growth in xenograft models of pediatric leukemias and solid tumors [4] and first preclinical studies also suggest effects in malignant rhabdoid tumors [30].

The effects of nuclear export inhibition on tumor growth are broad and it is unlikely that in rhabdoid tumor cells, only one single tumor suppressor pathway is affected. Nevertheless, our finding that inhibition of nuclear export restores nuclear localization and residual tumor suppressor function of truncated SMARCB1 protein suggests that a molecular subset of ATRT, i.e., cases harboring C-terminal *SMARCB1* mutations, might be especially responsive to treatment with selective nuclear export inhibitors. This concept could be further validated by taking into account *SMARCB1* mutational status and/or cytoplasmic SMARCB1 staining status within ongoing clinical trials (ClinicalTrials.gov Identifier: NCT02323880) and future studies with selective nuclear export inhibitors for the treatment of children with ATRT.

In conclusion, inhibition of nuclear export restores nuclear location and residual tumor suppressor function of truncated SMARCB1*.* Therapies aimed at preventing nuclear export of mutant SMARCB1 protein may represent a promising targeted therapy in ATRT harboring cytoplasmically localized truncated SMARCB1 proteins.

## Supplementary Information

Below is the link to the electronic supplementary material.

Supplementary file1 (XLSX 20 KB)** Supplemental Table 1: **Detailed clinical and molecular data of 102 ATRT samples.

## Data Availability

The data that support the findings of this study are available from the corresponding authors upon reasonable request.
